# The Sonic Hedgehog Signaling Pathway Induces Myopic Development by Activating Matrix Metalloproteinase (MMP)-2 in Guinea Pigs

**DOI:** 10.1371/journal.pone.0096952

**Published:** 2014-05-08

**Authors:** Minjie Chen, Yishan Qian, Jinhui Dai, Renyuan Chu

**Affiliations:** 1 Department of Ophthalmology, EENT Hospital, Fudan University, Shanghai, China; 2 Key Laboratory of Myopia, Ministry of Health, Shanghai, China; Massachusetts Eye & Ear Infirmary, Harvard Medical School, United States of America

## Abstract

**Purpose:**

To investigate whether the Sonic hedgehog (Shh) signaling induces myopic development by increasing the expression of matrix metalloproteinase (MMP)-2 in guinea pigs.

**Methods:**

A translucent diffuser was glued onto the right eye to induce form-deprivation myopia (FDM) in 10 guinea pigs. Four guinea pigs were served as a control group. The other 100 guinea pigs were subdivided into 5 groups (20 per group) and received a 10 µl intravitreal injection every 2 days for 4 times. Two groups were injected with 20 or 50 µg/ml Shh amino-terminal peptide (Shh-N) into the right eye and 0.1% bovine serum albumin into the other. FDM was induced in the right eyes of the three cyclopamine-treated groups and both eyes were injected with 50, 100, or 200 µg/ml cyclopamine. Retinoscopic refraction and eye dimensions were assessed on Day 14 of treatment. MMP-2 protein expression was determined in both scleras by western blotting.

**Results:**

Both concentrations of Shh-N stimulated myopic development and axial growth as compared with control eyes. Myopia and axial elongation were significantly greater in the 50 µg/ml than in the 20 µg/ml Shh-N group (*P*<0.001 and *P* = 0.0019, respectively). All three doses of cyclopamine significantly attenuated myopic development compared with the FDM group (*P*<0.0001). Cyclopamine at 100 or 200 µg/ml significantly reduced axial elongation compared with the FDM group (*P* = 0.044 and *P* = 0.001, respectively). FDM-induced myopia and axial elongation were significantly greater in the 50 µg/ml than in the 200 µg/ml cyclopamine group (*P*<0.0001 and *P* = 0.008, respectively). MMP-2 expression was significantly greater in Shh-N–treated eyes than in the control eyes, and was lower in the cyclopamine plus FDM groups than in the FDM group.

**Conclusions:**

The Shh signaling pathway induces myopic development by activating MMP-2 in guinea pigs.

## Introduction

Myopia is a major public health concern and there is striking evidence for a rapid increase in its prevalence in recent years [1]. Animal models have enabled significant advances in our understanding of the refractive development and the regulation of eye growth during myopic development. Animal models of myopia have revealed that visually guided eye growth is regulated by several retinal substances, including vasoactive intestinal peptide [2], dopamine [3], retinoic acid [4], early growth response gene-1 (also called ZENK in chickens) [5], and Sonic hedgehog (Shh) [6]–[8]. Moreover, current theories of refractive development acknowledge the pivotal role of the sclera in the control of eye size and in myopic development. Some modulators, including matrix metalloproteinase (MMP)-2, are upregulated in the sclera in experimental myopia [9],[10]. Thus, it has been speculated that there is a local retinoscleral signaling cascade that regulates the expression of modulators in the sclera and controls scleral remodeling during myopic development [11].

Identifying the chemical messengers that initiate and mediate these scleral changes is critical to further our understanding of the mechanisms underlying myopic development. However, most previous studies were observational in design, and suggested that abnormal expression of signals and modulators were involved in experimental myopia. In the absence of intervention studies and because of the potential interactions among different signaling pathways, it has not been possible to determine the exact relationship between the upstream (retina) and downstream (sclera) components of specific signaling pathways. Therefore, considering that many of the processes occurring in the extracellular matrix of the sclera of myopic eyes are controlled by specific growth factors and hormones in other fibrous matrices, we thought that evaluation of the Shh pathway would help us to understand the contribution of the retinoscleral signaling pathway to myopic development.

Hedgehog (hh) is a secreted protein that was originally identified in Drosophila, in which it plays a role in determining segment polarity. Seven hedgehog homologs have been identified in vertebrate species, with the best characterized being Shh. Shh signaling is mediated by two proteins: patched and smoothened. Shh plays a key role in many stages of embryonic eye development, including eye vesicle patterning, neuronal differentiation, optic stalk development, and axon guidance [12],[13]. Furthermore, Akamatsu et al. and Escaño et al. reported that in chick retinas [6],[7], Shh expression was increased in experimental myopia, suggesting that Shh may regulate the signaling cascade that leads to axial elongation and vitreous enlargement of the myopic eye. A more recent study revealed that the expression of Shh signaling factors was altered in mice with form-deprivation myopia (FDM), and the authors concluded that the Shh signaling pathway influences both FDM and the growth of eyes with normal visual input [8]. However, the exact downstream components of the Shh signaling and specific relationship between the downstream components and the Shh signaling pathway in the myopic development remain unknown. Additionally, several recent studies have suggested that Shh signaling is a critical regulator of the activities and/or expression of matrix metalloproteinase (MMP)-2 in cancer [14],[15]. It was also reported that the expression and activity of MMP-2 were enhanced by exogenous Shh and were inhibited by blocking Shh signaling with a Shh neutralizing antibody or cyclopamine in hepatocellular carcinoma samples [16]. Taken together, these results suggest that Shh might induce myopic development by enhancing the expression and activity of MMP-2 in animal models of myopia. Therefore, in this study, we up-regulated the Shh signals with exogenous Shh and down-regulated with cyclopamine to figure out the relationship between the Shh signal and myopic development as well as the relationship between the Shh signal and MMP-2 expression to understand the contribution of the retinoscleral signaling pathway in myopic development.

## Methods

### Animals

All experiments conformed to the Association for Research in Vision and Ophthalmology Statement for the Use of Animals in Ophthalmic and Vision Research, and were approved by the Committee for Animal Welfare of the EENT Hospital of Fudan University. Pigmented guinea pigs (*Cavia porcellus*, approximately 2 weeks of age) were obtained from the laboratory of Fudan University, China. The guinea pigs were reared under a 12 h light/dark cycle (lights on at 8 a.m.). The room temperature was maintained at 24°C. Water and food (guinea pig pellets, hay, and occasional fresh vegetables) were freely available.

### Effects of intravitreal Shh-N on refractive development, axial length, and MMP-2 expression

Fifty-four guinea pigs were randomly divided into four groups. A translucent diffuser (3 cm diameter) was glued onto the right eye to induce FDM in 10 guinea pigs (FDM). The diffuser was carefully attached to the fur around one eye with cyanoacrylic glue. The guinea pigs were checked three times per day to ensure the diffusers were in place. During refraction and biometric measurements, the diffusers were gently removed with forceps, which generally removed some fur surrounding the eye. Once refraction measurements were completed, the diffuser was re-affixed. Four age-matched untreated guinea pigs served as a control group.

Recombinant mouse Shh amino-terminal peptide (Shh-N) (R&D Systems, Minneapolis, MN, USA) was dissolved in saline and 0.1% bovine serum albumin (BSA) to final concentrations of 20 and 50 µg/ml. The right eye in each animal (*n* = 20) was injected with 10 µl of Shh-N solution (Table 1), while the left eye was injected with 0.1% BSA. Intravitreal injections were started immediately and were repeated every 2 days for a total of 4 times. Retinoscopic refraction and eye dimensions were determined before treatment and on Day 14 of treatment. Guinea pigs were anesthetized by an intramuscular injection of a solution containing ketamine (95 mg/ml) and xylazine (5 mg/ml) at a dose of 0.1 ml/100 g body weight. A drop of 0.4% oxybuprocaine hydrochloride (Santen Pharmaceuticals, Osaka, Japan) was applied for additional topical anesthesia. Lincomycin hydrochloride ophthalmic 2.5% solution (Sine Pharma Laboratory, Shanghai, China) was applied to the ocular surface before drug injection.

**Table 1:** Experimental groups and reasons for excluding guinea pigs treated with Shh-N.

Experimental groups			Reason for exclusion			
Groups	No.tested	No. included	Cataract	Retinal detachment	Death	Vitreous hemorrhage
Shh-N (20 µg/ml)	13	7	3	1	3	0
Shh-N (50 µg/ml)	12	8	3	0	4	1

Shh-N: Shh amino-terminal peptide.

Intravitreal injections were performed using a 30 G needle and a microinjector (Shanghai Meter Glass Factory, Shanghai, China), 0.5 mm posterior to the temporal limbus, with the needle angled towards the optic nerve until the tip of the needle was visualized in the center of the vitreous. Ofloxacin eye ointment (Santen Pharmaceuticals, Osaka, Japan) was applied after injection. Guinea pigs with severe cataract, vitreous hemorrhage, or retinal detachment were excluded (Table 1).

### Effects of intravitreal cyclopamine on refractive development, axial length, and MMP-2 expression in guinea pigs with FDM

Cyclopamine (Biomol, Plymouth Meeting, PA, USA) was dissolved in 45% 2-hydroxypropyl-*β*-cyclodextrin (Sigma, St. Louis, MO, USA) in phosphate-buffered saline by stirring for 1–2 h at 65°C to final concentrations of 50, 100, and 200 µg/ml. Sixty guinea pigs were randomly divided into three groups (*n* = 20 per group). FDM was induced in the right eye using a diffuser, as described above, while the left eye was used as a control. Both eyes in the three FDM groups were injected with 10 µl of cyclopamine (Table 2) immediately before occlusion and every 2 days for a total of 4 times, as described above. Retinoscopic refraction and eye dimensions were determined before and on Day 14 of treatment. Guinea pigs with severe cataract or endophthalmitis, and those that died were excluded from the study (Table 2).

**Table 2:** Experimental groups and reasons for excluding guinea pigs with FDM and treated with cyclopamine.

Experimental groups			Reason for exclusion		
Groups	No. tested	No. included	Cataract	Endophthalmitis	Death
FDM + Cyclo (50 µg/ml)	13	7	3	1	3
FDM + Cyclo (100 µg/ml)	12	8	4	0	4
FDM + Cyclo (200 µg/ml)	10	10	3	1	6

FDM: form-deprivation myopia; Cyclo: cyclopamine.

### Measurement of refraction and eye dimensions

Refractive state and eye dimensions were determined before and on Day 14 of treatment. Measurements were performed by researchers masked to the treatment group. Refraction was determined by streak retinoscopy with trial lenses in a dark room. At 1 h before retinoscopy, 1 drop of 0.5% tropicamide (Sanhe Pharmaceutical, Wuxi, China) was topically administered every 5 min for a total of 5 times to achieve complete pupil dilation. The refractive state was recorded as the mean refractions along the horizontal and vertical meridians.

A-scan ultrasonography (11 MHz; HiScan; Optikon S.p.A., Rome, Italy) was performed to measure the axial length of the eye, which included the anterior segment length (depth of the anterior chamber and the corneal thickness), the thickness of the crystalline lens, and the length of the vitreous chamber. The conducting velocity was 1540 m/s for measurements of the anterior segment and the vitreous chamber, and was 1645 m/s for measurement of the crystalline lens. Corneal anesthesia was achieved by topical application of 0.4% oxybuprocaine hydrochloride before ultrasound measurements. The ultrasound probe was placed directly on the cornea during measurements. The tip of the probe had a red light that was used to align the probe perpendicular to the corneal surface at the corneal apex. A genuine measurement was confirmed when clear traces of various components of the eye with consistent waves and amplitudes were obtained. Each axial component was calculated as the mean of the 10 repeated measurements in each eye.

### Western blotting

At the end of the experiment, the guinea pigs were sacrificed with an overdose of a solution containing ketamine (95 mg/ml) and xylazine (5 mg/ml). The eyeball was enucleated, and a punch of the posterior sclera was removed 1–2 mm nasal to the optic nerve with a 6 mm surgical trephine. The retina and choroid were carefully removed from the sclera. Scleral proteins were extracted by sonicating the tissue in ice-cold lysis buffer containing protease inhibitors. The primary antibody against MMP-2 was from Abcam (Cambridge, MA, USA) (0.5 µg/ml). The secondary antibody was from Jackson ImmunoResearch Laboratories (Baltimore, PA, USA). The primary antibody against glyceraldehyde phosphate dehydrogenase (GAPDH) was a gift from Miao Tong Biologic Science & Technology Co. Ltd (Shanghai, China).

### Pathology

Treated and untreated eyes were immersion-fixed in 3% glutaraldehyde/0.5% paraformaldehyde in 0.1 M phosphate buffer (pH 7.4), and then embedded in paraffin. The eyeball was cut into sections of 5 µm thick, stained with hematoxylin and eosin, and then observed under a microscope.

### Statistical analysis

All data are presented as the mean and standard error of the mean. For refractive states and ocular dimensions, data represent the difference between the right eye and the left eye. Refraction and axial length were compared between the right and left eyes using Student's paired *t* test or the Wilcoxon matched-pairs signed-rank test. Analysis of variance (ANOVA) or the Kruskal–Wallis test was used to test for difference in refraction or axial length among different groups, and the Bonferroni test was used to identify which pairs of treated groups were significantly different. The significance level was α = 0.05. All statistical tests were performed using Stata 11.0 (Stata Corp., College Station, TX, USA).

## Results

### Effects of intravitreal Shh-N on refractive development, axial length, and MMP-2 expression

The right eyes in the FDM and both Shh-treated groups exhibited significant myopia and axial elongation compared with the left contralateral eyes at the end of observation, whereas there were no differences between the left and right eyes in the control group (Table 3). Refraction was significantly different among the three treated groups (*P* = 0.0001). Myopia was significantly greater in the FDM and 50 µg/ml Shh group than in the 20 µg/ml Shh group (*P*<0.001). However, the refractive shift in the FDM group was not significantly different from that in the 50 µg/ml Shh group (*P* = 0.206). The relative axial elongation was also significantly different among the three treated groups (*P* = 0.0071). In particular, axial elongation was significantly greater in the 50 µg/ml Shh group than in the 20 µg/ml Shh group (*P* = 0.0019). The trend in the change of vitreous chamber length was consistent with that of axial length. Additionally, the right eyes in all groups showed similar changes in anterior chamber depth and length thickness relative to those in the left eyes at the end of the experiment (Table 3).

**Table 3:** Effects of intravitreal Shh-N administration on refraction and eye dimensions.

	FDM (*n* = 10)		Control (*n* = 4)		Shh-N (20 µg/ml) (*n* = 13)		Shh-N (50 µg/ml) (*n* = 12)	
	Difference	*P*	Difference	*P*	Difference	*P*	Difference	*P*
Refraction (D)	−5.13±1.73	<0.001	0.25±0.84	0.5943	−1.54±0.75	<0.001	−4.04±1.48	<0.001
ACD (mm)	−0.001±0.06	0.7344	−0.01±0.01	0.1817	0±0.01	1.0000	0.001±0.01	0.5637
Len thickness (mm)	0.02±0.04	0.2847	−0.01±0.02	0.4860	0.01±0.02	0.1682	0.01±0.01	0.0642
Axial length (mm)	0.11±0.04	<0.001	−0.01±0.02	0.7046	0.11±0.09	0.0014	0.14±0.03	<0.001
Vitreous length (mm)	0.10±0.07	0.0009	0.01±0.04	0.8130	0.1±0.09	0.0015	0.13±0.03	<0.001

Values represent the difference between the right and left eyes and are presented as the mean ± SD. FDM: form-deprivation myopia; ACD: anterior chamber depth; Shh-N: Shh amino-terminal peptide.

**Figure 1:**
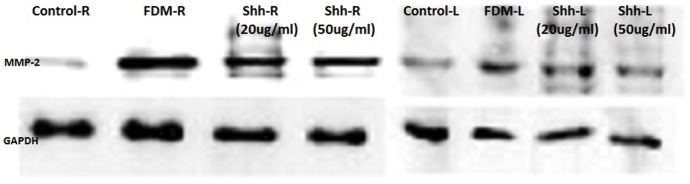
Western blotting analysis of MMP-2 protein expression in the sclera of guinea pigs following intravitreal Shh-N injection. FDM: form-deprivation myopia; R: right eye; L: left eye; Shh-N: Shh amino-terminal peptide;MMP-2: matrix metalloproteinase-2.

At the end of the experiment, MMP-2 protein expression in the right eyes of the FDM and both Shh-treated groups was significantly greater than that in the left eyes and in the control group. Moreover, MMP-2 protein expression in the right eyes was greater in the FDM group than in both Shh-treated groups. However, MMP-2 protein expression was not significantly different between the 20 µg/ml and 50 µg/ml Shh groups (Figure 1).

### Effects of intravitreal cyclopamine on refractive development, axial length, and MMP-2 expression in guinea pigs with FDM

All three doses of cyclopamine significantly attenuated the induction of myopia as compared with the FDM group (*P*<0.0001). In particular, 200 µg/ml cyclopamine almost completely eliminated the induction of myopia (*P* = 0.1773). Although the refractive shift in the 100 µg/ml cyclopamine group was not significantly different from that in the 50 µg/ml (*P* = 0.079) and the 200 µg/ml (*P* = 0.236) cyclopamine groups, the extent of myopia was significantly greater in the 50 µg/ml group than in the 200 µg/ml group (*P*<0.0001) (Table 4). In all cyclopamine groups, axial length was greater in the right eyes than in the left eyes, while the differences were not statistically significant between the FDM group and the FDM plus 50 µg/ml cyclopamine group (*P* = 1.0000). Meanwhile, 100 and 200 µg/ml cyclopamine significantly reduced axial elongation compared with the FDM group (*P* = 0.044 and *P* = 0.001, respectively). Although relative axial elongation in the 100 µg/ml group was not significantly different from that in the 50 and 200 µg/ml groups, axial length was significantly shorter in the 200 µg/ml group than in the 50 µg/ml group (*P* = 0.008). Cyclopamine significantly reduced axial growth in the left eyes by 0.02±0.03 mm in the 50 µg/ml group (*P* = 0.0331), by 0.06±0.06 mm in the 100 µg/ml group (*P* = 0.0058), and by 0.04±0.1 mm in the 200 µg/ml group (*P* = 0.1397). This reduction in growth was not significantly different among the three groups (*P* = 0.2992). The trend in the change of vitreous chamber length was consistent with that of axial length. At the end of the experiment, the right eyes in all groups showed similar changes in anterior chamber depth and length thickness relative to those in the left eyes.

**Table 4:** Effects of intravitreal cyclopamine administration and FDM on refraction and eye dimensions.

	FDM (*n* = 10)		Control (*n* = 4)		FDM + Cyclo (50 µg/ml) (*n* = 13)		FDM + Cyclo (100 µg/ml) (*n* = 13)		FDM + Cyclo (200 µg/ml) (*n* = 13)	
	Difference	*P*	Difference	*P*	Difference	*P*	Difference	*P*	Difference	*P*
Refraction (D)	−5.13±1.73	<0.001	0.25±0.84	0.5943	−2.69±1.15	<0.001	−1.46±0.91	0.0002	0.38±0.81	0.1773
ACD (mm)	−0.001±0.06	0.7344	−0.01±0.01	0.1817	0.002±0.02	0.6410	−0.01±0.01	0.0819	−0.002±0.01	0.6193
Lens thickness (mm)	0.02±0.04	0.2847	−0.01±0.02	0.4860	0.004±0.02	0.4331	−0.003±0.01	0.5715	−0.004±0.03	0.7109
Axial length (mm)	0.11±0.04	<0.001	−0.01±0.02	0.7046	0.09±0.05	<0.001	0.06±0.04	0.0006	0.08±0.07	0.0053
Vitreous length (mm)	0.10±0.07	0.0009	0.01±0.04	0.8130	0.09±0.05	<0.001	0.07±0.05	0.0007	0.08±0.09	0.0153

Values represent the difference between the right and left eyes and are presented as the mean ± SD. FDM: form-deprivation myopia; ACD: anterior chamber depth; Cyclo: cyclopamine.

**Figure 2:**
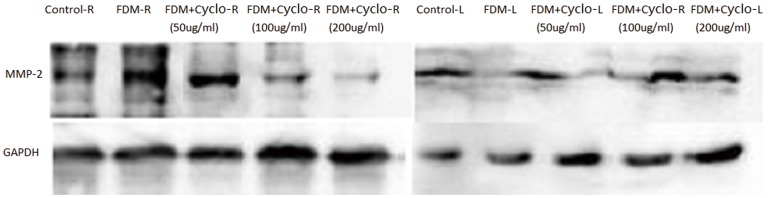
Western blotting analysis of MMP-2 protein expression in the sclera of guinea pigs following FDM and intravitreal cyclopamine injection. FDM: form-deprivation myopia; Cyclo: cyclopamine; R: right eye; L: left eye; MMP-2: matrix metalloproteinase-2.

MMP-2 protein expression in the right eyes at Day 14 was significantly greater in the FDM group and in the FDM plus 50 µg/ml cyclopamine group than in the left eyes and the control group. Although MMP-2 protein expression was not significantly different between the FDM group and the FDM plus 50 µg/ml cyclopamine group, MMP-2 expression was greater in the 50 µg/ml group than in the 100 or 200 µg/ml groups (Figure 2).

### Pathologic findings

**Figure 3:**
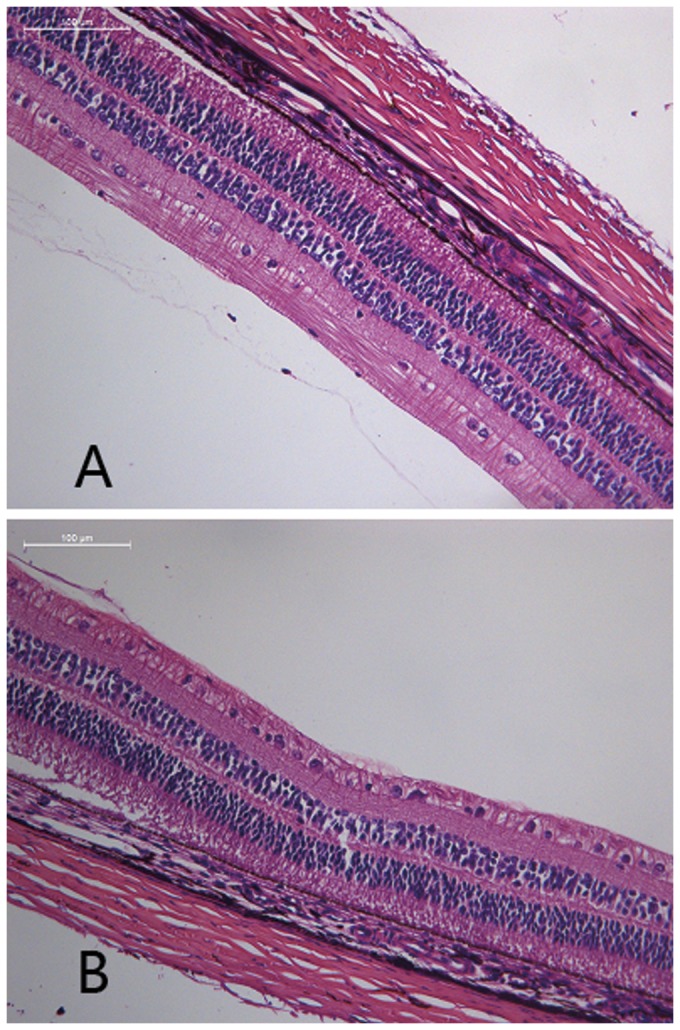
Pathologic examination of eye sections stained with hematoxylin and eosin. The retina of eyes treated with Shh-N (B) were morphologically indistinguishable from control eyes treated with solvent alone (A). Shh-N: Shh amino-terminal peptide.

The retina of occluded and non-occluded eyes treated with either Shh-N or cyclopamine were morphologically indistinguishable from control eyes treated with solvent alone (Figure 3).

## Discussion

The present study is the first to show that Shh regulates myopic development in guinea pigs. Intraocular administration of Shh-N induced the progression of experimental myopia. Moreover, myopic development and axial elongation were significantly greater in eyes treated with 50 µg/ml Shh-N than in eyes treated with 20 µg/ml Shh-N, which indicates that Shh-N stimulates growth and myopic refractive shift in a dose-dependent manner. Meanwhile, cyclopamine, an antagonist of the Shh signaling pathway, reduced the myopic refractive error and inhibited FDM-induced axial elongation, and the highest dose of cyclopamine (200 µg/ml) virtually eliminated myopic development. We also found that refractive shift and axial growth were significantly different between eyes treated with 200 and 50 µg/ml cyclopamine. The dose-dependent pro-myopic effects of Shh-N and the anti-myopic effects of cyclopamine provide evidence that Shh is involved in the regulation of refractive development in guinea pigs, consistent with the results of a previous study in mice [8]. However, mice are unlikely to exhibit accommodation because their eyes lack ciliary muscles [17]. Furthermore, their small eyes make it difficult to measure refraction and axial length, thereby resulting in increased measurement error and reduced reliability of the results [8]. Thus, guinea pigs may be a more suitable animal model for studying human myopia because it is a precocial species born with a well-developed visual system and shows similar scleral changes to humans during myopic development.

The current study showed that myopic development and axial growth induced by Shh-N were caused mainly by lengthening of the vitreous chamber, which supports the hypothesis that scleral remodeling is involved in myopic development [11]. Upregulation of MMP-2 expression was reported to be essential for scleral remodeling in experimental myopia in previous studies [9],[10]. Therefore, we expected to see an increase in MMP-2 protein expression in Shh-N–treated eyes relative to control eyes, and a decrease in its expression in the cyclopamine plus FDM eyes than in FDM eyes without cyclopamine treatment. Furthermore, intraocular administration of agonists and antagonists of the Shh signaling pathway dose-dependently altered MMP-2 expression in our study. Taken together, these results suggest that Shh signaling induced the myopic shift and axial elongation in guinea pigs by enhancing MMP-2 expression. With the determination of the exact relationship between the upstream of Shh signaling in the retina and downstream of MMP-2 in the scleral, we help in understanding the contribution of the retinoscleral signaling pathway to myopic development for the first time.

Generally, tissue inhibitors of metalloproteinase (TIMP) regulate the activity of MMP-2 and other MMPs. The balance between MMP-2 and TIMP-2 is critical in maintaining normal metabolic activity in various tissues, including the sclera. Previous findings suggest that increased TIMP-2 levels may be particularly important in regulating the increased activation of MMP-2 during myopic development [11]. Although we only determined MMP-2 expression in the current study, another study focusing on liver cancer found no change in TIMP-1 and TIMP-2 protein expression after the administration of Shh-N, anti-Shh, or cyclopamine, which suggests that TIMP-1 and TIMP-2 are not directly involved in Shh-mediated induction of MMP-2 expression [16]. Further studies are needed to identify the role of TIMP-2 in Shh-mediated experimental myopia. For another, our data have led to the investigation of Shh and MMP-2 as putative components of the signaling pathway between the retina and sclera. However, myopic development and axial elongation are complex processes involving multiple changes in various signals and associated factors. Considering our knowledge of MMP-2, particularly its role in the metabolic activities of the sclera, these changes are thought to represent the final common pathway in the retinoscleral signal that regulates eye growth. In particular, several studies have presented evidence that the phosphoinositide 3-kinase (PI3K)–Akt pathway can regulate MMP production and activation in Shh-induced carcinoma migration and invasion [16],[18]. The PI3K–Akt pathway was also reported to be activated in plus lens-treated myopia while a PI3K inhibitor attenuated the effects of insulin in minus lens-treated myopia in chicks [19]. In addition, researchers studying the source of the signal driving these changes in scleral fibroblasts found that isolated retinal pigment epithelial (RPE) cells could influence the growth patterns of cultured scleral chondrocytes [20]. The authors proposed that RPE cells contain or express factors that can mediate the retinoscleral signaling pathway [20]. Other studies have revealed that the activities of RPE cells are controlled via the PI3K–AKT pathway [21]-[23]. In addition, the Shh pathway was reported to inhibit transdifferentiation of RPE [24]. Taken together, these data suggest that the PI3K–AKT might be the upstream pathway controlling MMP-2 expression in Shh-induced experimental myopia and that PRE cells might be the source of the signal. Nevertheless, further studies are needed to examine the roles of other branches of the Shh signaling pathway in myopic development.

In conclusion, our results indicate that the Shh signaling pathway contributes to the refractive shift and axial elongation in guinea pigs. Our findings also highlight the potential role of MMP-2 in Shh-induced myopic development. Thus, these data have revealed a novel signaling mechanism involved in myopic development and a potential molecular target for preventing or treating myopia.
